# Fructosamine and Hemoglobin A1c Correlations in HIV-Infected Adults in Routine Clinical Care: Impact of Anemia and Albumin Levels

**DOI:** 10.1155/2015/478750

**Published:** 2015-07-26

**Authors:** Luisa Duran, Carla Rodriguez, Dan Drozd, Robin M. Nance, J. A. Chris Delaney, Greer Burkholder, Michael J. Mugavero, James H. Willig, Amy H. Warriner, Paul K. Crane, Ben E. Atkinson, Robert D. Harrington, Shireesha Dhanireddy, Michael S. Saag, Mari M. Kitahata, Heidi M. Crane

**Affiliations:** ^1^University of Washington, Seattle, WA 98195, USA; ^2^University of Alabama at Birmingham, Birmingham, AL 35294, USA

## Abstract

Fructosamine is an alternative method to hemoglobin A1c (HbA1c) for determining average glycemia. However, its use has not been extensively evaluated in persons living with HIV (PLWH). We examined the relationship between HbA1c and fructosamine values, specifically focusing on anemia (which can affect HbA1c) and albumin as a marker of liver disease. We included 345 PLWH from two sites. We examined Spearman rank correlations between fructosamine and HbA1c and performed linear test for trends to compare fructosamine and HbA1c correlations by hemoglobin and albumin quartiles. We examined discrepant individuals with values elevated only on one test. We found a correlation of 0.70 between fructosamine and HbA1c levels. Trend tests for correlations between fructosamine and HbA1c were significant for both albumin (*p* = 0.05) and hemoglobin (*p* = 0.01) with the lowest correlations in the lowest hemoglobin quartile. We identified participants with unremarkable HbA1c values but elevated fructosamine values. These discrepant individuals had lower mean hemoglobin levels than those elevated by both tests. We demonstrated a large correlation between HbA1c and fructosamine across a range of hemoglobin and albumin levels. There were discrepant cases particularly among those with lower hemoglobin levels. Future studies are needed to clarify the use of fructosamine for diabetes management in PWLH.

## 1. Introduction

The dramatic decline in HIV-related mortality since the introduction of potent antiretroviral therapy (ART) [1–3] has been accompanied by an increase in metabolic derangements including abnormalities in glucose metabolism such as type 2 diabetes mellitus [4–6]. The pathogenesis of these conditions is likely multifactorial and includes the direct effects of HIV itself [7] and metabolic complications associated with antiretroviral therapy (ART) [4, 5].

Hemoglobin A1c (HbA1c) is an index of mean glycemia and is used to diagnose and monitor diabetes [8, 9]. However, low HbA1c values may be an artifact related to factors that shorten the lifespan of erythrocytes [9, 10] and studies suggest that HbA1c can underestimate average glucose measures in PLWH due to subclinical hemolysis associated with medications including dapsone, ribavirin, and trimethoprim-sulfamethoxazole and other conditions that shorten the lifespan of red blood cells such as chronic renal disease [11–13]. Many studies evaluating HbA1c among PLWH considered small numbers of participants [11, 14], were conducted early in the ART treatment era when regimens were different [11], and targeted PLWH with diabetes [11, 13, 14] limiting their generalizability to the current treatment era and across the full spectrum of HbA1c values.

Measuring fructosamine is an alternative method for determining average glycemia over a two- to three-week period [10] and may be particularly useful in settings where altered erythrocyte turnover interferes with HbA1c values [9]. Unlike HbA1c, fructosamine measurements reflect glycated serum proteins that are not affected by red blood cell half-life [10, 15]. In contrast, fructosamine measurements may be altered in conditions associated with low concentrations of albumin and plasma proteins such as nephrotic syndrome or liver disease particularly cirrhosis [10, 15] which is common among PLWH [16]. The use of fructosamine to evaluate average glycemia has not been rigorously evaluated in PLWH although it is increasingly used in clinical practice for non-HIV infected adults [10, 15]. Fructosamine may be a quick, simple, affordable alternative to HbA1c that could help improve diabetes care in PLWH, especially in those patients with anemia or diminished red blood cell survival.

We conducted this study to evaluate associations between fructosamine and HbA1c in PLWH across the spectrum of glucose metabolism from no diabetes to poorly controlled diabetes. We examined whether these associations were similar among those with low hemoglobin levels and low albumin levels.

## 2. Methods

### 2.1. Study Design and Setting

This retrospective cross-sectional study was conducted among patients from the Centers for AIDS Research Network of Integrated Clinical Systems (CNICS). CNICS is an ongoing longitudinal observational cohort collaboration of PLWH receiving primary care after January 1, 1995, to the present at eight clinical sites [17]. PLWH from two clinical sites, the University of Washington and the University of Alabama at Birmingham, were included in these analyses.

### 2.2. Study Participants

HIV-infected adults (19 years of age or older) with both fructosamine and HbA1c measures assessed within 30 days of each other during routine care were eligible for this study. We also included adults who had stored serum or plasma drawn within 30 days of an existing HbA1c measured as part of routine care. PLWH known to be on dialysis or to be pregnant were excluded. CNICS sites have local institutional review board approval.

### 2.3. Data Sources

The CNICS data repository captures longitudinal data on PLWH and served as the primary data source for this study. The CNICS data repository integrates comprehensive clinical data from all outpatient and inpatient encounters including standardized HIV-related information collected at enrollment. Demographic, clinical, laboratory, and medication data are obtained from each site's electronic health record and other institutional data sources. Information regarding patients' age, race/ethnicity, sex, HIV transmission factor, albumin and protein levels, markers of anemia (hemoglobin), CD4 cell count, and HIV-1 viral load was included in our analyses. For other laboratory measures, the values closest to the date of the fructosamine values were used. As an example for albumin, the median difference between the albumin and fructosamine values was 0 days, interquartile range −1 day to 0 days. All participants had an HbA1c value drawn as part of clinical care; we considered values ≥ 6.5% to be elevated.

### 2.4. Measurement of Fructosamine

A total of 126 fructosamine values were measured as part of routine clinical care on the same day as or within 30 days of HbA1c values using a colorimetric assay (Beckman Coulter AU640). We measured fructosamine from an additional 219 frozen serum samples drawn within 30 days of an HbA1c value to ensure a broad range of clinical characteristics and diabetes status. While HbA1c and fructosamine values could differ by as many as 30 days, the median difference between fructosamine and HbA1c values was 0 days, interquartile range 0 to 0 days. The quantitative determination of fructosamine on human serum was performed on a Roche automated clinical chemistry analyzer (Modular P). This colorimetric assay is based on the ability of ketoamines to reduce nitrotetrazolium blue (NBT) to formazan in an alkaline solution. The rate of formation of formazan is directly proportional to the concentration of fructosamine. A reference range of 205 to 285 *μ*mol/L is considered to be normal with values above 285 *μ*mol/L elevated.

### 2.5. Statistical Analysis

We compared demographic and clinical characteristics of PLWH with normal and elevated HbA1c (≥6.5%) values and normal and elevated fructosamine (≥285 *μ*mol/L) values using Chi squared tests. We examined discrepant participants defined as those who had elevated values by one measure (HbA1c or fructosamine) but not the other. We calculated Spearman rank correlation coefficients to describe the correlation between HbA1c and fructosamine measures and divided participants into quartiles based on hemoglobin and albumin levels to examine correlations within these quartiles. We also examined how these correlations differed across a range of HbA1c values (<5.7, 5.7–6.4, and ≥6.5%) [18] as a measure of diabetes severity categories. We performed a linear test for trend to compare correlations between fructosamine and HbA1c values by quartiles for hemoglobin and albumin and by diabetes severity categories. As an example, a significant trend test across groups defined by hemoglobin would imply that the correlations between fructosamine and HbA1c were significantly increasing or decreasing across the hemoglobin quartiles.

Methods to correct fructosamine values for low albumin levels have been previously suggested [10, 19, 20]. We repeated analyses using a correction factor where fructosamine values were adjusted for albumin levels as follows:

 corrected fructosamine (mmol/L) = measured fructosamine + 0.03 (40 − serum albumin g/L) [20].

We plotted HbA1c and fructosamine values for those with normal and abnormal hemoglobin and albumin levels and included a LOWESS curve or fitted line for each plot based on those with normal hemoglobin and albumin levels defined as the top 3 quartiles for each.

We conducted linear regression analyses to examine the ability of HbA1c to predict fructosamine and repeated models adjusting for age, race, and sex. We also repeated models adjusting for age, race, sex, body morphology index (BMI) category, current CD4 cell count, and HIV transmission risk category. We used results from linear regression analyses to calculate predicted HbA1c from fructosamine values among those in the bottom quartile of hemoglobin, the group of greatest concern for problematic HbA1c measures, and compared predicted and measured HbA1c values. All analyses were conducted using SAS version 9.2, Cary, NC, or STATA version 13, College Station, TX.

## 3. Results

We examined fructosamine and HbA1c levels in 345 participants (188 from University of Alabama at Birmingham, 157 from University of Washington). Demographic and clinical characteristics are presented in Table 1, stratified by both fructosamine and HbA1c values. The study population was predominantly men (77%) and the mean age was 48 years (standard deviation [SD] 9). Almost half (48%) had a current CD4 count ≥500 cells/mm^3^, and 85% were on nucleoside reverse transcriptase inhibitors (NRTIs), with tenofovir/lamivudine or tenofovir/emtricitabine occurring most commonly (61%). Regimens containing didanosine and stavudine were being used by <3% and 1%, respectively. Obesity (body mass index ≥ 30 kg/m^2^) was present in 37%. We found excellent agreement between HbA1c and fructosamine with an overall correlation of 0.70.

When we divided participants into quartiles by albumin levels, the correlation between HbA1c and fructosamine was strongly maintained throughout all four quartiles as shown in Table 2. Similarly, this correlation was maintained throughout all quartiles of hemoglobin. As expected, correlations were the lowest among those with the lowest quartile of hemoglobin. Tests of trend, suggesting a difference in correlations by quartile, were significant for both albumin (*p* = 0.046) and hemoglobin (*p* = 0.01). When we used standardized fructosamine values by correcting for albumin, results were similar (Supplementary Table 1 in Supplementary Material available online at http://dx.doi.org/10.1155/2015/478750). Tests of trend for correlations between albumin-corrected fructosamine values and HbA1c by quartile were significant for both albumin (*p* = 0.05) and hemoglobin (*p* = 0.01).

We repeated correlations by disease status as measured by HbA1c categories (Supplementary Table 2). Correlations between fructosamine and HbA1c were much lower among patients with HbA1c < 5.7, compared with 5.7–6.4, or ≥ 6.5 with a significant test for trend (*p* < 0.001).

**Figure 1:**
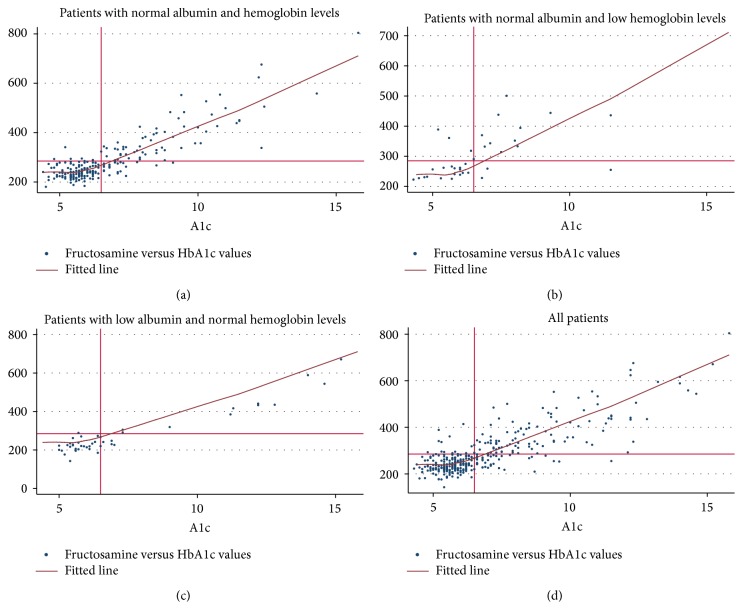
The plots show HbA1c and fructosamine values from our sample. Superimposed on the graph are the lines at the threshold between normal and abnormal fructosamine values (fructosamine = 285) and normal and abnormal HbA1c values (HbA1c = 6.5). (a) plots HbA1c and fructosamine values from patients with normal albumin and hemoglobin levels defined as being in the top 3 quartiles for hemoglobin and albumin levels. Superimposed on the graph is a LOWESS curve, which is a smoothed curve showing the central tendency at each value among those with normal albumin and hemoglobin levels. Correlation between HbA1c and fructosamine = 0.72. (b) plots HbA1c and fructosamine values from patients with low hemoglobin levels (lowest quartile) and normal albumin levels. The LOWESS line is from Figure 1(a) for those with normal hemoglobin and albumin levels. Correlation between HbA1c and fructosamine = 0.63. (c) plots HbA1c and fructosamine values from patients with low albumin levels (lowest quartile) and normal hemoglobin levels. The LOWESS line is from Figure 1(a) for those with normal hemoglobin and albumin levels. Correlation between HbA1c and fructosamine = 0.75. (d) plots HbA1c and fructosamine values from all patients. The LOWESS line is from Figure 1(a) for those with normal hemoglobin and albumin levels. Correlation between HbA1c and fructosamine = 0.70.

Figures 1(a)–1(d) plot HbA1c and fructosamine values with thresholds between normal and abnormal HbA1c and fructosamine values superimposed on the plots.  Figure 1(a) includes only those with normal hemoglobin and albumin levels defined as the top 3 quartiles for each. We superimposed a LOWESS curve, which is a fitted line or smoothed curve showing the central tendency at each value, on the plot shown in Figure 1(a). The LOWESS curve and most of the data points are close to a diagonal line, indicating excellent linear agreement between the two measures. The high level of agreement is also supported by the large number of points in the top right (both fructosamine and HbA1c values are abnormal) and bottom left (both fructosamine and HbA1c values are normal) quadrants. The correlation between fructosamine and HbA1c in this subset with normal hemoglobin and albumin levels was 0.72.  Figure 1(b) plots HbA1c and fructosamine values among those with normal albumin levels (top 3 quartiles of albumin) but low hemoglobin levels (bottom quartile of hemoglobin). This subset includes people for whom there may be concern that HbA1c measures may be less accurate (would underestimate degree of hyperglycemia). We superimposed the LOWESS curve used in Figure 1(a) on the data shown in Figure 1(b). The correlation between fructosamine and HbA1c in this subset was 0.63.  Figure 1(b) shows more values and scatter above the LOWESS curve than below, consistent with higher levels as measured by fructosamine than by HbA1c in this group with low hemoglobin levels. In contrast, Figure 1(c) includes those with normal hemoglobin values (top three quartiles of hemoglobin) but low albumin values (bottom quartile of albumin). This subset includes people for whom there may be concern that fructosamine may be less accurate (would underestimate degree of hyperglycemia). The correlation between fructosamine and HbA1c in this subset was 0.75. Plotted values are predominantly lower than the LOWESS curve derived from people with normal values of both hemoglobin and albumin, demonstrating higher HbA1c values and lower fructosamine values in this group with low albumin levels. Finally, Figure 1(d) plots HbA1c and fructosamine values among all participants (correlation 0.70 as described above).

We identified 13 individuals with an unremarkable HbA1c (<6.5) who had elevated fructosamine values (top left quadrant of Figure 1(d)) who would not have been identified as having elevated glycemia had we relied exclusively on HbA1c values. These individuals had slightly lower mean hemoglobin levels (13.1 g/dL, SD 1.8) than people who had elevated glycemia as measured by both fructosamine and HbA1c (13.6 g/dL, SD 2.1) but similar albumin levels (3.9 g/dL, SD 0.5 versus 3.9 g/dL, SD 0.5). Another 39 individuals had elevated HbA1c levels (≥6.5) but did not have elevated fructosamine levels (bottom right quadrant of Figure 1(d)). These individuals had similar mean hemoglobin levels compared to those who had elevated glycemia as measured by both tests (13.7 g/dL, SD 1.9 versus 13.6 g/dL, SD 2.1) but slightly lower albumin levels (3.7 g/dL, SD 0.5 versus 3.9 g/dL, SD 0.5) and would not have been identified as having elevated glycemia had we relied exclusively on fructosamine levels.

We conducted linear regression analyses among those in the top 3 quartiles of albumin and hemoglobin with fructosamine values as the dependent variable and found that each one-point higher HbA1c value was associated with a mean fructosamine value 42.0 *μ*mol/L higher (*p* < 0.001). Note the intercept for this model was 3.2, *p* = 0.8, so it can be ignored in evaluating this relationship. The relationship between HbA1c and fructosamine was essentially unchanged in models that are also adjusted for age, race, and sex (41.8 *μ*mol/L, *p* < 0.001). Based on this linear regression analysis, we calculated predicted HbA1c values based on measured fructosamine values among those in the bottom quartile of hemoglobin with normal albumin levels (top three quartiles of albumin; see Figure 1(b)). Measured HbA1c values were on average 0.5 (SD 1.6) lower than HbA1c values calculated from measured fructosamine in this group.

## 4. Discussion

This study demonstrated a high correlation between HbA1c and fructosamine values in PLWH in routine clinical care (0.70). HbA1c values were lower among those with low hemoglobin values, and fructosamine values were lower among those with low albumin levels. A small subset of patients (*N* = 13, 4%) had discordant results such that their HbA1c was not elevated but their fructosamine was elevated, and another small subset of patients (*N* = 39, 11%) had elevated HbA1c but not elevated fructosamine levels. Measured HbA1c values were 0.5 lower than calculated HbA1c values among those with lower hemoglobin levels suggesting that these individuals may have measured HbA1c values that are artificially low. These data are very helpful in prioritizing the use of these tests in clinical care for PLWH.

### 4.1. Fructosamine and HIV

An earlier case-control study of 100 PLWH and 200 HIV-uninfected controls found that, among PLWH with diabetes or hyperglycemia, HbA1c underestimated glucose values and that nucleoside reverse transcriptase inhibitor (NRTI) use was strongly associated with this discordance while fructosamine was less likely to underestimate glucose and was not impacted by NRTIs [13]. While useful, this study was limited to patients with DM or significant hyperglycemia and excluded those with anemia and other comorbidities limiting generalizability. We found that, particularly among individuals with lower hemoglobin levels, measured HbA1c values were lower than calculated HbA1c values derived from fructosamine measurements, suggesting that the measured HbA1c values underestimated glycemia. However, this was not limited to just those with severe anemia. Since many PLWH are treated with medications or affected by conditions that disrupt the erythrocyte lifespan, fructosamine may be an appropriate alternative to HbA1c for assessing glucose control in individuals with lower hemoglobin levels. A key difference is the timeframes of the two measures. The shorter timeframe of fructosamine could be considered a limitation for some purposes. However, the faster response to changes can also be considered a strength because it allows more rapid information on whether changes are effective, since HbA1c takes much longer time to reflect responses to treatment changes. While more information is needed to further identify exactly which PLWH would be most likely to benefit from fructosamine versus HbA1c testing in clinical care, this study suggests that particularly those with low hemoglobin values might benefit from fructosamine rather than HbA1c values.

### 4.2. Participants with Discrepant Results Using the Different Measures of Glycemia

From a clinical standpoint, patients with normal HbA1c measures but elevated fructosamine values would be of particular interest. In our study, these individuals had lower mean hemoglobin levels compared to people who had elevated glycemia as measured by both HbA1c and fructosamine. In these individuals, differences between measured HbA1c and HbA1c derived from fructosamine values were small on average (0.5 HbA1c units) and were associated with lower levels of hemoglobin but not severe anemia. Similarly, we identified participants with elevated HbA1c values but without elevated fructosamine values. These people had slightly lower albumin levels than those with elevated values by both tests, but the differences were small suggesting it may be exceedingly difficult to identify which PLWH will be discrepant and/or should receive one test versus the other.

## 5. Strengths and Limitations

Strengths of this study include the focus on associations between HbA1c and fructosamine across a range of glucose abnormalities. We examined how these associations might differ among those with various albumin and hemoglobin levels, which may be particularly relevant in PLWH given the high prevalence of liver disease and erythrocyte turnover [16, 21, 22]. To ensure a broad distribution of diabetes status including patients with prediabetes or no diabetes as well as demographic and clinical diversity, we included paired fructosamine and HbA1c values ordered as part of clinical care at two sites and also results from frozen CNICS specimen repository samples from another 219 patients.

A limitation of this study is that a majority of patients had an HbA1c in the 5–8% range with very few observations from people with extremely high HbA1c values. We did not have continuous glucose monitoring on patients to determine which measure was more accurate. We examined people with discrepant results using dichotomous cutoffs, such as 6.5 for HbA1c. These useful but necessarily limited cut-points could miss persons with DM and falsely diagnose others without DM [23, 24]. This approach also necessarily focuses attention on only one part of the spectrum. We did not have data to evaluate intraperson variability in fructosamine measures. Finally, given that HbA1c and fructosamine measure glycemia over different time periods, some differences in estimated glycemia from these two values may reflect changing glycemia over time.

## 6. Conclusions

This study provides new evidence that HbA1c and fructosamine are strongly correlated in PLWH across a range of hemoglobin and albumin levels. With the evolving emphasis on using HbA1c for diabetes diagnosis and not just monitoring treatment, understanding the characteristics of HbA1c and fructosamine testing across the entire spectrum of diabetes status is increasingly important. This study provides information regarding the use of fructosamine in PLWH with varied levels of albumin and hemoglobin. Overall, our findings suggest that HbA1c or fructosamine can be used interchangeably in the majority of PLWH. Nevertheless, there are particular subgroups of PLWH who may benefit from using fructosamine to measure glycemia in preference to HbA1c. While low hemoglobin levels may be a useful marker, it is difficult to clearly identify those individuals who would benefit from one test over the other, as they are not just limited to those with severe anemia or liver disease. Future studies are needed to better define the clinical applicability of fructosamine testing in the clinical care of PWLH and to identify those individuals who are better served with fructosamine versus HbA1c testing.

## Supplementary Material

Supplement Table 1 demonstrates the correlations between HbA1c and fructosamine. Note that for supplement Table 1, these values of fructosamine are corrected for albumin levels. Correlations are presented by quartile for hemoglobin and albumin level.
Supplement Table 2 demonstrates the correlations between HbA1c and fructosamine by diabetes severity levels.

## Figures and Tables

**Table 1:** Demographic and clinical characteristics (*N* = 345).

Characteristics	Fructosamine			HbA1c			Total
	<285	≥285	*p* value	<6.5	≥6.5	*p* value	*N* = 345
	*N* = 230	*N* = 115		*N* = 204	*N* = 141		
Sex							
Male	185 (80)	79 (69)		167 (82)	97 (69)		264 (77)
Female	45 (20)	36 (31)	0.02	37 (18)	44 (31)	0.005	81 (23)
Race							
White	129 (56)	51 (44)		116 (57)	64 (45)		180 (52)
Black	83 (36)	59 (51)		72 (35)	70 (50)		142 (41)
Hispanic	8 (3)	1 (<1)		8 (4)	1 (<1)		9 (3)
Other	10 (4)	4 (3)	0.04	8 (4)	6 (4)	0.02	14 (4)
HIV transmission risk factor							
MSM	115 (50)	59 (51)		104 (51)	70 (50)		174 (50)
IDU	56 (24)	14 (12)		52 (25)	18 (13)		70 (20)
Heterosexual	57 (25)	41 (36)		47 (23)	51 (36)		98 (28)
Other	2 (<1)	1 (<1)	0.03	1 (<1)	2 (1)	0.006	3 (<1)
CD4 cell count (current)							
≥500	119 (52)	46 (40)		94 (46)	71 (50)		165 (48)
350–499	40 (17)	27 (23)		40 (20)	27 (19)		67 (19)
200–349	40 (17)	25 (22)		41 (20)	24 (17)		65 (19)
<200	31 (13)	17 (15)	0.2	29 (14)	19 (13)	0.9	48 (14)
CD4 cell count (nadir)							
≥500	54 (23)	17 (15)		43 (21)	28 (20)		71 (21)
350–499	36 (16)	14 (12)		31 (15)	19 (13)		50 (14)
200–349	58 (25)	24 (21)		52 (25)	30 (21)		82 (24)
<200	82 (36)	60 (52)	0.03	78 (38)	64 (45)	0.6	142 (41)
HIV-1 viral load							
<10,000	199 (87)	101 (88)		177 (87)	123 (87)		300 (87)
10,000–99,999	16 (7)	11 (10)		14 (7)	13 (9)		27 (8)
≥100,000	15 (7)	3 (3)	0.2	13 (6)	5 (4)	0.4	18 (5)
Body mass index							
<25	63 (27)	35 (30)		64 (31)	34 (24)		98 (28)
25–29	85 (37)	33 (29)		74 (36)	44 (31)		118 (34)
≥30	82 (36)	47 (41)	0.3	66 (32)	63 (45)	0.06	129 (37)
Fructosamine level							
<285	—	—		191 (94)	39 (28)		230 (67)
≥285				13 (6)	102 (72)	<0.001	115 (33)
HbA1c level							
<6.5	191 (83)	13 (11)		—	—		204 (59)
≥6.5	39 (17)	102 (89)	<0.001				141 (41)

**Table 2:** Correlation between HbA1c and fructosamine by albumin and hemoglobin quartile.

	Percentile	0	25	50	75	100
Albumin	g/dL	1.3	3.6	3.9	4.2	5.1
	Spearman's rho		0.75	0.74	0.77	0.66

Hemoglobin	g/dL	6.7	12.7	14	15.2	17.9
	Spearman's rho		0.63	0.68	0.73	0.75

## References

[1] Palella F. J., Delaney K. M., Moorman A. C. (1998). Declining morbidity and mortality among patients with advanced human immunodeficiency virus infection. HIV Outpatient Study Investigators. *The New England Journal of Medicine*.

[2] Murphy E. L., Collier A. C., Kalish L. A. (2001). Highly active antiretroviral therapy decreases mortality and morbidity in patients with advanced HIV disease. *Annals of Internal Medicine*.

[3] Hogg R. S., Heath K. V., Yip B. (1998). Improved survival among HIV-infected individuals following initiation of antiretroviral therapy. *Journal of the American Medical Association*.

[4] Carr A., Samaras K., Thorisdottir A., Kaufmann G. R., Chisholm D. J., Cooper D. A. (1999). Diagnosis, prediction, and natural course of HIV-1 protease-inhibitor-associated lipodystrophy, hyperlipidaemia, and diabetes mellitus: a cohort study. *The Lancet*.

[5] Behrens G., Dejam A., Schmidt H. (1999). Impaired glucose tolerance, beta cell function and lipid metabolism in HIV patients under treatment with protease inhibitors. *AIDS*.

[6] Tien P. C., Schneider M. F., Cole S. R. (2007). Antiretroviral therapy exposure and incidence of diabetes mellitus in the Women's Interagency HIV Study. *AIDS*.

[7] El-Sadr W. M., Mullin C. M., Carr A. (2005). Effects of HIV disease on lipid, glucose and insulin levels: results from a large antiretroviral-naïve cohort. *HIV Medicine*.

[8] The International Expert Committee (2009). International Expert Committee report on the role of the A1C assay in the diagnosis of diabetes. *Diabetes Care*.

[9] Sacks D. B., Arnold M., Bakris G. L. (2011). Guidelines and recommendations for laboratory analysis in the diagnosis and management of diabetes mellitus. *Diabetes Care*.

[10] Wright L. A.-C., Hirsch I. B. (2012). The challenge of the use of glycemic biomarkers in diabetes: reflecting on hemoglobin A1C, 1,5-anhydroglucitol, and the glycated proteins fructosamine and glycated albumin. *Diabetes Spectrum*.

[11] Polgreen P. M., Putz D., Stapleton J. T. (2003). Inaccurate glycosylated hemoglobin A1C measurements in human immunodeficiency virus-positive patients with diabetes mellitus. *Clinical Infectious Diseases*.

[12] Diop M.-E., Bastard J.-P., Meunier N. (2006). Inappropriately low glycated hemoglobin values and hemolysis in HIV-infected patients. *AIDS Research and Human Retroviruses*.

[13] Kim P. S., Woods C., Georgoff P. (2009). A1C underestimates glycemia in HIV infection. *Diabetes Care*.

[14] Kim S.-Y., Friedmann P., Seth A., Fleckman A. M. (2014). Monitoring HIV-infected patients with diabetes: hemoglobin A1c, fructosamine, or glucose?. *Clinical Medicine Insights: Endocrinology and Diabetes*.

[15] Armbruster D. A. (1987). Fructosamine: structure, analysis, and clinical usefulness. *Clinical Chemistry*.

[16] Weber R., Sabin C. A., Friis-Moller N. (2006). Liver-related deaths in persons infected with the human immunodeficiency virus: the D:A:D study. *Archives of Internal Medicine*.

[17] Kitahata M. M., Rodriguez B., Haubrich R. (2008). Cohort profile: the centers for AIDS research network of integrated clinical systems. *International Journal of Epidemiology*.

[18] (2011). Standards of medical care in diabetes—2012. *Diabetes Care*.

[19] Van Dieijen-Visser M. P., Seynaeve C., Brombacher P. J. (1986). Influence of variations in albumin or total-protein concentration on serum fructosamine concentration. *Clinical Chemistry*.

[20] Howey J. E. A., Browning M. C. K., Fraser C. G. (1987). Assay of serum fructosamine that minimizes standardization and matrix problems: use to assess components of biological variation. *Clinical Chemistry*.

[21] Kellerman S. E., Hanson D. L., McNaghten A. D., Fleming P. L. (2003). Prevalence of chronic hepatitis B and incidence of acute hepatitis B infection in human immunodeficiency virus-infected subjects. *The Journal of Infectious Diseases*.

[22] Graham C. S., Baden L. R., Yu E. (2001). Influence of human immunodeficiency virus infection on the course of hepatitis C virus infection: a meta-analysis. *Clinical Infectious Diseases*.

[23] Cohen R. M., Lindsell C. J. (2012). When the blood glucose and the HbA_1c_ don't match: turning uncertainty into opportunity. *Diabetes Care*.

[24] Cohen R. M., Haggerty S., Herman W. H. (2010). HbA1c for the diagnosis of diabetes and prediabetes: is it time for a mid-course correction?. *Journal of Clinical Endocrinology and Metabolism*.

