# Supplementary material for: Fructosamine and Hemoglobin A1c Correlations in HIV-Infected Adults in Routine Clinical Care: Impact of Anemia and Albumin Levels
Source: AIDS Res Treat. 2015 Jul 26;2015:478750. doi: 10.1155/2015/478750 (PMC4529884; doi:10.1155/2015/478750)
Supplement: Supplementary file 1 — Supplement Table 1 demonstrates the correlations between HbA1c and fructosamine. Note that for supplement Table 1, these values of fructosamine are corrected for albumin levels. Correlations are presented by quartile for hemoglobin and albumin level. Supplement Table 2 demonstrates the correlations between HbA1c and fructosamine by diabetes severity levels. [file 478750.f1.pdf]

Supplement Table 1. Correlation between HbA1c and Albumin corrected Fructosamine by Albumin and Hemoglobin quartile

	Percentile	0	25	50	75	100
Albumin	g/dL	1.3	3.6	3.9	4.2	5.1
	Spearman's rho		0.75	0.74	0.77	0.67
Hemoglobin	g/dL	6.7	12.7	14	15.2	17.9
	Spearman's rho		0.64	0.68	0.73	0.75

corrected fructosamine (mmol/l)=(measured fructosamine + 0.03 (40-serum abumin g/l), mmol/L[20].

Supplement Table 2. Correlation between HbA1c and fructosamine by diabetes severity level as measured by HbA1c

HbA1c (%)	<5.7	5.7-6.4	≥6.5
Spearman's rho	-0.08	0.16	0.68
